# Development of an Acute and Highly Pathogenic Nonhuman Primate Model of Nipah Virus Infection

**DOI:** 10.1371/journal.pone.0010690

**Published:** 2010-05-18

**Authors:** Thomas W. Geisbert, Kathleen M. Daddario-DiCaprio, Andrew C. Hickey, Mark A. Smith, Yee-Peng Chan, Lin-Fa Wang, Joseph J. Mattapallil, Joan B. Geisbert, Katharine N. Bossart, Christopher C. Broder

**Affiliations:** 1 National Emerging Infectious Diseases Laboratories Institute, Boston University School of Medicine, Boston, Massachusetts, United States of America; 2 Department of Microbiology, Boston University School of Medicine, Boston, Massachusetts, United States of America; 3 Department of Medicine, Boston University School of Medicine, Boston, Massachusetts, United States of America; 4 Department of Microbiology and Immunology, Uniformed Services University, Bethesda, Maryland, United States of America; 5 Pathology Division, United States Army Medical Research Institute of Infectious Diseases, Fort Detrick, Frederick, Maryland, United States of America; 6 Australian Animal Health Laboratory, Livestock Industries, Australian Commonwealth Scientific and Research Organization, Geelong, Victoria, Australia; National Institutes of Health, United States of America

## Abstract

Nipah virus (NiV) is an enigmatic emerging pathogen that causes severe and often fatal neurologic and/or respiratory disease in both animals and humans. Amongst people, case fatality rates range between 40 and 75 percent and there are no vaccines or treatments approved for human use. Guinea pigs, hamsters, cats, ferrets, pigs and most recently squirrel monkeys (New World monkey) have been evaluated as animal models of human NiV infection, and with the exception of the ferret, no model recapitulates all aspects of NiV-mediated disease seen in humans. To identify a more viable nonhuman primate (NHP) model, we examined the pathogenesis of NiV in African green monkeys (AGM). Exposure of eight monkeys to NiV produced a severe systemic infection in all eight animals with seven of the animals succumbing to infection. Viral RNA was detected in the plasma of challenged animals and occurred in two of three subjects as a peak between days 7 and 21, providing the first clear demonstration of plasma-associated viremia in NiV experimentally infected animals and suggested a progressive infection that seeded multiple organs simultaneously from the initial site of virus replication. Unlike the cat, hamster and squirrel monkey models of NiV infection, severe respiratory pathology, neurological disease and generalized vasculitis all manifested in NiV-infected AGMs, providing an accurate reflection of what is observed in NiV-infected humans. Our findings demonstrate the first consistent and highly pathogenic NHP model of NiV infection, providing a new and critical platform in the evaluation and licensure of either passive and active immunization or therapeutic strategies for human use.

## Introduction

Nipah virus (NiV) is a recently recognized viral zoonotic pathogen of the family *Paramyxoviridae* that is distinguished by its ability to cause fatal disease in both animal and human hosts. NiV along with Hendra virus (HeV) comprise the new genus *Henipavirus* (reviewed [1], [2]). NiV was first identified during an outbreak of severe encephalitis in Malaysia and Singapore in 1998–99 that involved hundreds of people and more than 100 deaths, with pigs serving as the intermediate amplifying host [3], [4]. Since 1998 there have been more than a dozen recognized occurrences of human NiV infection, primarily in Bangladesh and India (reviewed [5]) with the two most recent outbreaks in March 2008 [6] and January 2010 [7]. In the majority of subsequent spillover events, the mortality rate among humans has been higher (∼75%) along with evidence of multiple rounds of person-to-person transmission [8].

Several species of fruit bats of the *Pteropus* genus (flying foxes) appear to be the principle natural reservoirs of both NiV and HeV (reviewed [5]) but serological evidence of NiV or Nipah-like virus infection has recently been reported in several additional frugivorous and insectivorous bats [9], [10]. NiV has been isolated from bat urine and partially eaten fruit [11], [12] and direct transmission of NiV from flying foxes to humans from contaminated food sources has been suggested [13], [14]. The Centers for Disease Control and Prevention (CDC) and the National Institute of Allergy and Infectious Diseases (NIAID) have classified NiV as a select agent with the potential for causing significant morbidity and mortality in humans and major economic and public health impacts. Consequently, work with live virus requires Biosafety Level 4 (BSL-4) containment.

Host cell infection by NiV requires two membrane-anchored envelope glycoproteins; the attachment glycoprotein (G) which binds receptor and the fusion (F) glycoprotein which facilitates virus-host cell membrane fusion [2]. NiV G lacks hemagglutinin and neuraminidase activities and F is a typical class I fusion glycoprotein [15]. Recently, ephrin-B2 and ephrin-B3 ligands were identified as receptors employed by the henipaviruses for infection [16], [17], [18], [19]. For paramyxoviruses, the majority of virus-neutralizing antibody is directed towards these two envelope glycoproteins with the attachment glycoprotein serving as the dominant target antigen [20], [21], [22]. Indeed, recombinant soluble G from HeV and NiV has been successfully employed as a subunit vaccine in a NiV challenge model in the cat [23], [24], and evidence of passive antibody protection against NiV and HeV challenge in the hamster has been reported [25], [26], [27]. More recently a cross reactive, neutralizing and fully human monoclonal antibody has been shown to be an effective post-exposure therapeutic against NiV in a new ferret model [28]. However, there are currently no vaccines or therapeutic modalities approved for the prevention or treatment of henipavirus infections in humans.

The development and characterization of suitable animal models for henipavirus infection is essential for understanding their pathogenic characteristics and mechanisms as well as fulfilling the critical needs for the *in vivo* evaluation of potential antiviral modalities for human use. To date, guinea pigs, golden hamsters, cats, ferrets and pigs have been evaluated as experimental animal models of human NiV infection (reviewed[5], [29], [30]). The restriction of live henipavirus experimentation to BSL-4 containment has significantly hampered the development of henipavirus countermeasures in a rapid and systematic fashion. In addition, the U.S. Food and Drug Administration (FDA) has implemented the Animal Efficacy Rule which came into effect in 2002. This rule specifically applies to the development of therapeutic products when human efficacy studies are not possible or ethical, such as is often the case with highly virulent emerging pathogens like NiV. Essentially, this rule allows for the evaluation of vaccines or therapeutics using data derived from studies carried out in at least two animal models. Although the ferret seems to be the most accurate animal model developed thus far, the licensure of any antiviral therapeutic for NiV will likely also require an evaluation of NiV pathogenesis in nonhuman primates. However, there has been little experimentation directed towards the evaluation of henipavirus infection outcomes in nonhuman primates. Here we describe a new animal model of acute NiV infection and associated disease in the African green monkey (AGM).

## Results

### Nipah Virus Infection and Pathogenesis in African Green Monkeys

There is no information presently available on the sequence homology between the human and African green monkey (AGM) (*Chlorocebus aethiops*) ephrin-B2 or ephrin-B3 receptors. However, we reasoned that the human and AGM ephrins should be quite similar given the high amino acid sequence homology across a variety of vertebrate species. While none of the henipaviruses have been specifically adapted to a simian host or for usage of a simian ephrin receptor protein NiV and HeV replicate to very high titers (>10^7^) in Vero cells which are derived from AGMs. We first performed a challenge study in three AGMs (Subjects 1–3) with doses ranging from 7.0×10^3^ to 1.3×10^6^ plaque forming units (pfu) of NiV by intratracheal (i.t.) and oral delivery. In this initial experiment, the monkeys appeared highly susceptible to acute disease caused by exposure to NiV (**Table 1**). All three animals became severely ill with symptoms highly consistent with human NiV infection and disease [31], [32], [33], [34], [35].

**Table 1:** Clinical description and outcome of Nipah virus challenged monkeys.

Subject No.	Sex	Dose (pfu)	Route	Clinical illness	Clinical and gross pathology
1	Male	1.3×10^6^	i.t., oral	Depression, lethargy, fever, loss of appetite, severe dyspnea, open-mouth breathing. Animal euthanized on day 9.	Thrombocytopenia; excess blood-tinged pleural fluid; severely inflated, enlarged lungs with multifocal areas of congestion and hemorrhage; copious sanguinous frothy fluid exuding from nose and mouth; hemorrhages on mucosal surface of urinary bladder.
2	Male	7.0×10^3^	i.t., oral	Depression, lethargy, loss of appetite from day 4–17, dyspnea, open-mouth breathing, nausea, lymphadenopathy, ecchymotic rash at venipuncture site, muscle twitches, behavioral changes. Recovered after long convalescence.	Mild thrombocytopenia.
3	Female	8.1×10^4^	i.t., oral	Depression, lethargy, fever, loss of appetite, severe dyspnea, open-mouth breathing. X-ray showed pleural effusions. Nausea at end stages. Animal succumbed on day 11.	Excess blood-tinged pleural fluid; severely inflated, enlarged lungs with multifocal areas of congestion and hemorrhage; petechial to ecchymotic hemorrhages on mucosal surface of urinary bladder; apparent meningitis.
4	Male	6.5×10^4^	i.t.	Depression, lethargy, fever, loss of appetite, open-mouth breathing. X-ray showed pleural effusions. Animal euthanized on day 10.	Mild thrombocytopenia; rectal bleeding; excess blood-tinged pleural fluid; severely inflated, enlarged lungs with multifocal areas of congestion and hemorrhage; hemorrhages on mucosal surface of urinary bladder.
5	Male	5.9×10^4^	i.t.	Depression, lethargy, loss of appetite, open-mouth breathing. X-ray showed pleural effusions. Animal euthanized on day 11.	Thrombocytopenia; excess blood-tinged pleural fluid; severely inflated, enlarged lungs with multifocal areas of congestion and hemorrhage.
6	Male	2.3×10^4^	i.t.	Depression, lethargy, fever, loss of appetite, open-mouth breathing, loss of balance. X-ray showed pleural effusions. Animal succumbed on day 9.	Thrombocytopenia; excess blood-tinged pleural fluid; severely inflated, enlarged lungs with multifocal areas of congestion and hemorrhage; hemorrhages on mucosal surface of urinary bladder.
7	Male	7.0×10^3^	i.t.	Depression, lethargy, loss of appetite, open-mouth breathing. X-ray showed pleural effusions. Animal euthanized on day 9.	Thrombocytopenia; rectal bleeding; excess blood-tinged pleural fluid; severely inflated, enlarged lungs with multifocal areas of congestion and hemorrhage; hemorrhages on mucosal surface of urinary bladder.
8	Male	2.5×10^3^	i.t.	Depression, lethargy, loss of appetite, open-mouth breathing. X-ray showed pleural effusions. Animal succumbed on day 12.	Thrombocytopenia; excess blood-tinged pleural fluid; severely inflated, enlarged lungs with multifocal areas of congestion and hemorrhage; sanguinous frothy fluid exuding from nose and mouth; hemorrhages on mucosal surface of urinary bladder; apparent meningitis.

i.t., intratracheal; pfu, plaque forming unit.

**Figure 1:**
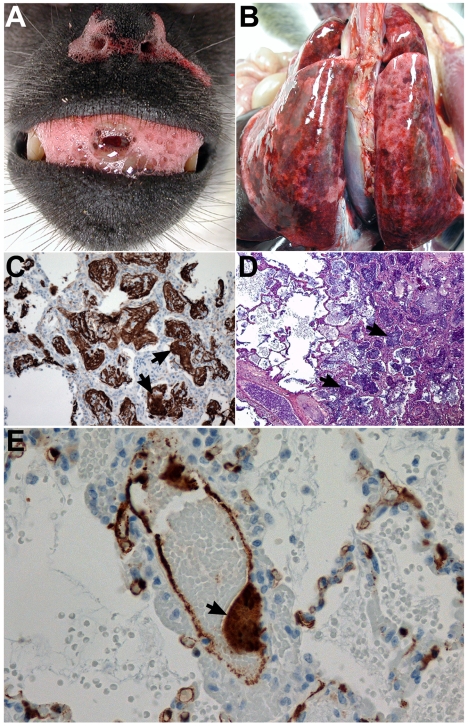
NiV pathogenesis in the African green monkey. Panels A, B, D, and E; Subject 1 euthanized on day 9 after i.t. and oral challenge with 1.3×10^6^ pfu of NiV. Panel C; Subject 3 that succumbed on day 11 after i.t. and oral challenge to 8.1×10^4^ pfu of NiV. (A) Sanguinuous fluid/froth exuding from nares and mouth. (B) Severely enlarged lungs; multifocal areas of congestion and hemorrhage. (C) Lung, right diaphragmatic lobe by immunohistochemical stain showing abundance of polymerized fibrin in and around alveolar spaces (arrows); 40× magnification. (D) Lung, right diaphragmatic lobe by PTAH stain showing abundance of polymerized fibrin in and around alveolar spaces (arrows); 100× magnification. (E) Localization of NiV antigen by immunohistochemical stain within a lung blood vessel with endothelial syncytia (arrow) and scattered foci of immunopositive cells (brown) abundant in alveolar septae; 400× magnification.

**Figure 2:**
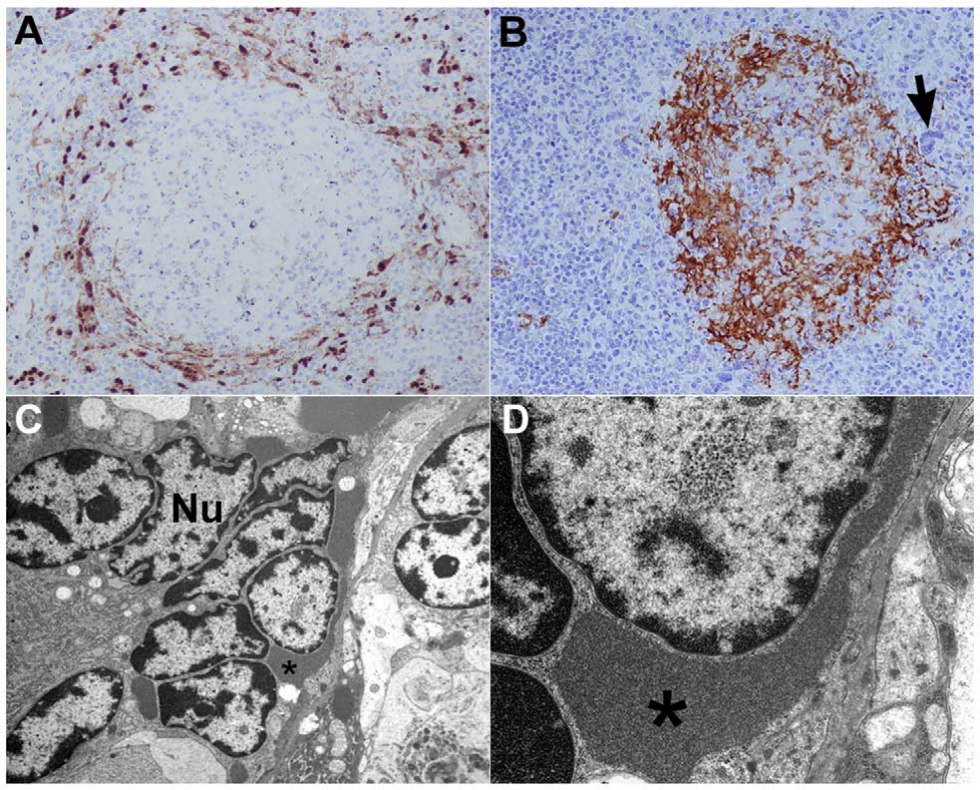
NiV infection and pathogenesis in the spleen. Panels A–D; Subject 1 that succumbed on day 9 after i.t. and oral challenge with 1.3×10^6^ pfu of NiV. (A) Localization of NiV by immunohistochemistry; 20× magnification. Scattered foci of immunopositive cells (brown) abundant in the parafollicular area. Note severe lymphocytolysis of follicle. (B) Localization of fibrin by immunohistochemistry showing abundance of fibrin (brown) in and around follicle from different section than panel A; 20× magnification. A multinucleate giant cell is evident in parafollicular area (arrow). (C) Transmission electron micrograph of spleen multinucleate giant cell 4,000× magnification, Nu, nucleus and asterisk indicates prominent intracytoplasmic paramyxoviral inclusions; and D: 20,000× magnification of asterisk indicated area shown in panel C.

**Figure 3:**
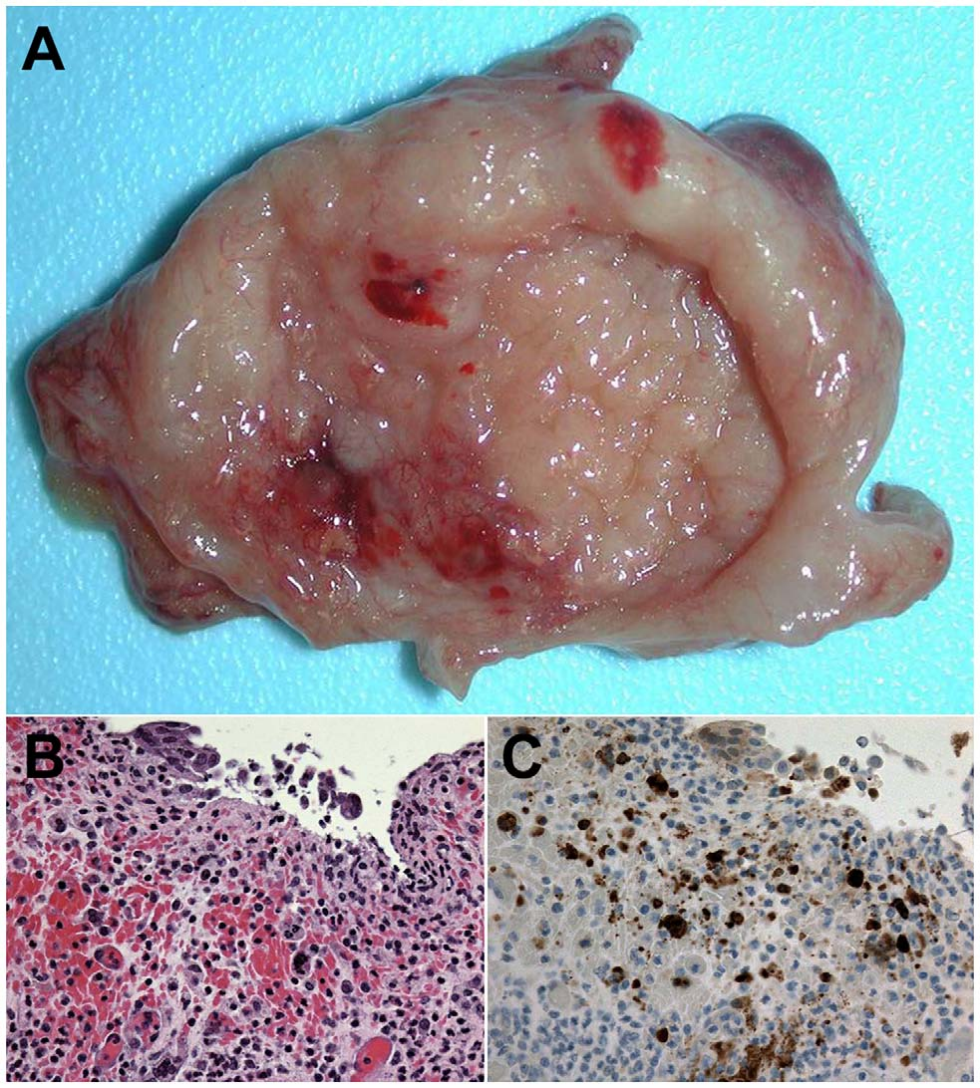
NiV infection and pathogenesis in the urinary bladder. Subject 3 that succumbed on day 11 after i.t. and oral exposure to 8.1×10^4^ pfu of NiV. (A) Petechial to ecchymotic hemorrhages on mucosal surface of urinary bladder of animal of animal at necropsy. (B) Bladder ulcer showing inflammation and hemorrhage by H&E staining; 400× magnification. (C) Bladder ulcer by immunohistochemistry staining of NiV antigen; 400× magnification.

Based on these initial findings, a second experiment was carried out in five AGMs (Subjects 4–8). NiV was inoculated i.t. and doses ranged from 2.5×10^3^ to 6.5×10^4^ pfu (**Table 1**). Together, these data indicated that NiV lethality in AGMs occurred within 9 to 12 days after virus infection with an i.t. or i.t and oral exposure with doses ranging from ∼2.5×10^3^ pfu to ∼1.3×10^6^ pfu of NiV (**Table 1**). All animals in these susceptibility studies (Subjects 1–8) developed a severe acute-respiratory-distress-syndrome (ARDS)-like disease, associated with copious amounts of sanguinous fluid and froth (**Figure 1A**). Gross pathological examination revealed severely inflated and enlarged lungs with multifocal areas of congestion and hemorrhage (**Figure 1B**). Immunohistochemical and histopathological examination showed significant amounts of polymerized fibrin and NiV antigen (**Figure 1C** and **1D**). Endothelial syncytial cells were particularly prominent in tissues of most animals and vasculitis and its sequelae were the most common lesions observed. NiV antigen was present in endothelial and arterial smooth muscle cells in most tissues examined with lung appearing to be the most severely affected organ (**Figure 1E**). Syncytial cells were also prominent in a variety of other tissues including spleen, a hallmark feature of these paramyxoviruses and also seen in naturally and experimentally infected animals (**Figure 2**) [24], [36], [37]. NiV is also known for its appearance and shedding in the urine of infected animals including bats, and the bladders of infected monkeys were involved showing inflammation and hemorrhage and high levels of NiV antigen (**Figure 3**). Immunohistochemical and histopathological analysis revealed the presence of NiV antigen, predominantly in endothelial cells and smooth muscle cells, along with associated pathology in a wide of variety tissues, including the tongue, salivary gland, larynx and trachea, heart, gall bladder, kidneys, sex organs, pituitary and adrenal glands, stomach, small and large intestine, and skeletal muscles (data not shown).

**Figure 4:**
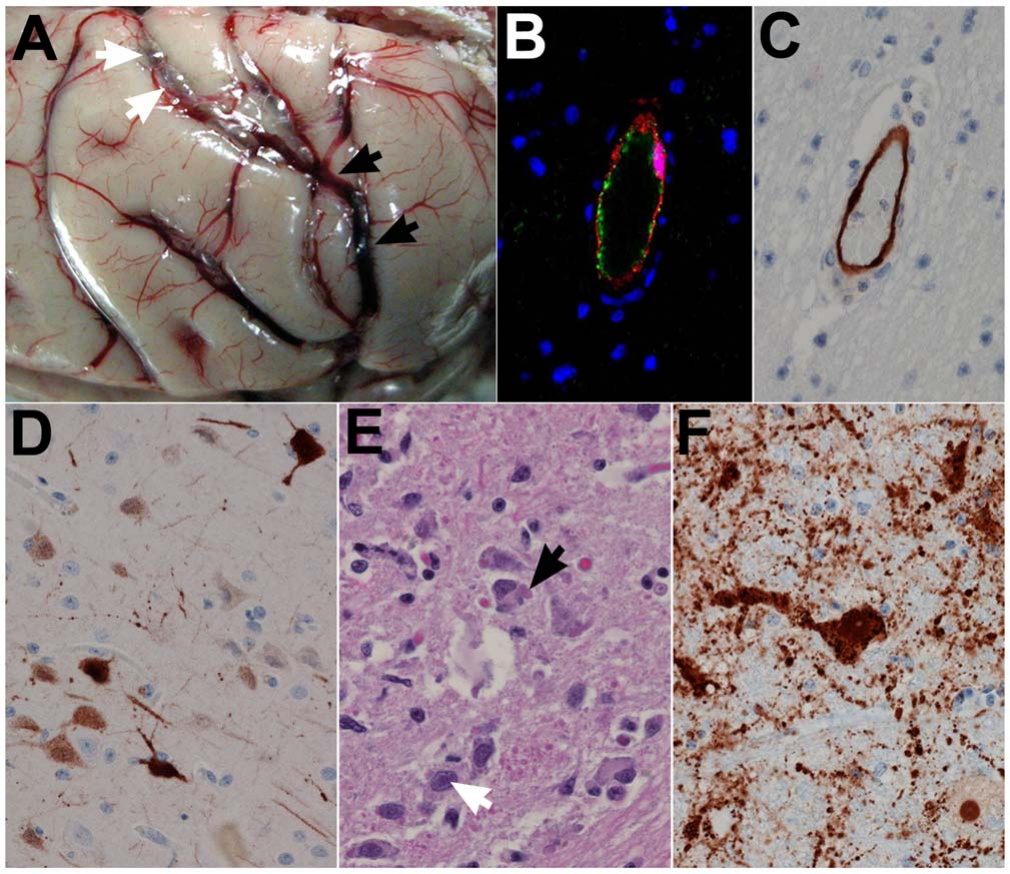
NiV infection and pathogenesis in the brain. Subject 3 that succumbed on day 11 after i.t. and oral exposure to 8.1×10^4^ pfu of NiV. (A) Brain, severe congestion (black arrows) with possible meningeal hemorrhage; fluid (white arrows) suggests mild to moderate meningeal edema; (B) brain, cerebrum, double labeling for an endothelial cell marker (von Willebrand's factor) (green) and NiV antigen (red) showing co-localization of NiV with the endothelium. Immuofluorescence method with DAPI counterstain; 400× magnification. Subject 6 that succumbed on day 10 after i.t. challenge with 2.3×10^4^ pfu of NiV; (C) brain, cerebrum (corpus striatum), localization of NiV antigen by immunohistochemistry, immunopositive cells (brown) associated with the endothelium; 400× magnification. Subject 8 that succumbed on day 12 after i.t. challenge with 2.5×10^3^ pfu of NiV; (D) brain, cerebrum (frontal cortex), localization of NiV antigen by immunohistochemistry, strong cytoplasmic and nuclear immunopositive neurons and processes. (E) Subject 6 brain stem and neurons with intranuclear inclusion bodies (white arrow) and intracytoplasmic inclusion bodies (black arrow), H&E staining; 400× magnification. Subject 5 that succumbed on day 11 after i.t. exposure to 5.9×10^4^ pfu of NiV; (E) brain stem immunohistochemistry staining of NiV antigen in same area as in (D) with antigen detected in neuron cell body and axon as well as surrounding cells; 400× magnification.

**Figure 5:**
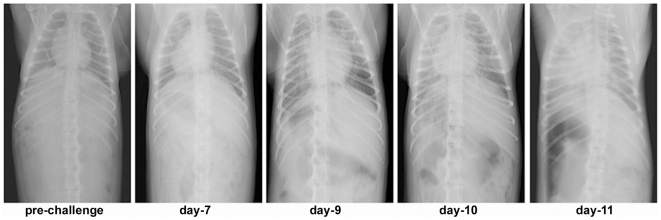
Progressive NiV pathogenesis in the African green monkey analyzed by X-ray autoradiography. Subject 3 that succumbed on day 11 after i.t. and oral exposure to 8.1×10^4^ pfu of NiV. X-ray images of chest, days 3 to 11 showing progressive lung pathology. Day 7, evidence of developing pneumonia or congestion. At day 10 and 11 congestion and pneumonia with infiltrates on the lung fields. These are the first X-ray images of severe lung pathology induced by a BSL-4 restricted viral pathogen.

In addition and of significant importance, most animals showed evidence of neurologic disease (**Table 1** and **Figure 4**). Severe congestion with evidence of meningeal hemorrhaging and edema was observed (**Figure 4A**). NiV antigen was detected in endothelial cells in brain of all animals in this study (Subjects 1, 3–8), example shown in **Figure 4B** and **4C**. In addition, most animals also showed NiV infection of neurons (**Figure 4D–F**), often widespread in the brainstem. Further, because of the observed unique respiratory involvement of the acute NiV infection in the monkeys, we sought approval for bringing in a portable X-ray device into the BSL-4 and subsequently carried out a series of X-ray images on one animal with signs of acute NiV disease (Subject 3; see **Table 1**). Here, changes in radiographs were evident by day 7 when ventrodorsal view showed developing pneumonia or congestion. At day 10 and 11 congestion and pneumonia with infiltrates on the lung fields were evident. At day 11 the heart appeared larger and rounder suggestive of pericardial effusion (**Figure 5**).

A single animal exposed to a low dose of ∼7×10^3^ pfu of NiV (Subject 2) became severely ill on day 4 and remained clinically severely ill until day 18 at which time the animal began to recover. Together, these data indicated that NiV lethality in AGMs occurred within 9 to 12 days after virus infection with an i.t. or i.t and oral exposure with doses ranging from ∼2.5×10^3^ pfu to ∼1.3×10^6^ pfu of NiV (**Table 1**).

### Nipah Viral Loads and Tissue Tropism in African Green Monkeys

**Figure 6:**
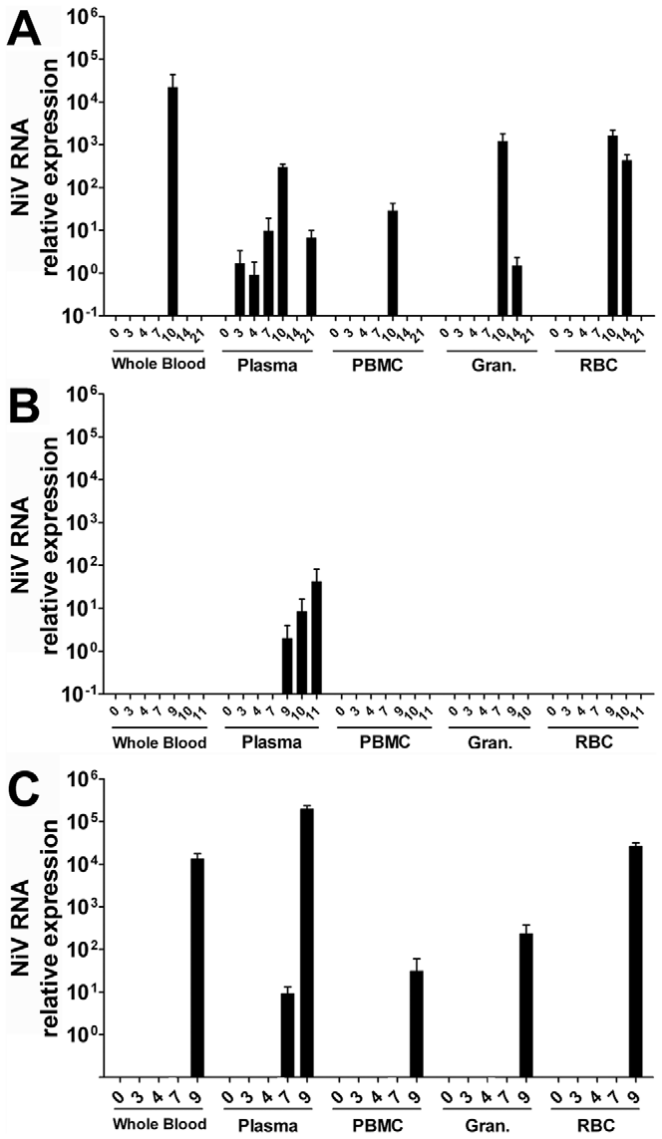
The normalized relative quantity of NiV RNA detected among blood samples from NiV-infected AGMs. Taqman RT-PCR was used to determine the relative NiV RNA level among samples of whole blood or blood components separated by Ficoll density centrifugation as described in the methods. (A) Low challenge dose, Subject 2; (B) Medium challenge dose, Subject 3; and (C) high challenge dose Subject 1.

To examine the extent of virus replication and tissue tropism among monkeys challenged with NiV, the relative quantity of the NiV RNA was measured in blood as well as swabs obtained from pharyngeal, rectal, and vaginal (where appropriate) surfaces sampled the course of infection. In addition, multiple tissues were harvested from NiV-challenged monkeys at the time of necropsy and the relative quantity of NiV RNA was measured. The relative quantity of NiV RNA was determined utilizing a Taqman probe that targets the NiV nucleoprotein (N) gene and detects cDNA derived from both genomic and positive sense RNA [23]. **Figure 6** shows a comparison of the NiV RNA levels detected at various times post infection from a series of whole blood samples and blood components collected during the initial AGM NiV challenge study. NiV RNA detection corresponded closely with the onset of clinical illness and the progression of the disease. Viral RNA was detected in the plasma of all challenged animals and is the first clear demonstration of plasma-associated viremia in NiV experimentally infected animals (**Figure 6**). Virus was also isolated from some plasma and whole blood samples when available (**Table 2**). Whole blood, plasma, and PBMC were also sampled periodically over a 12 day period following NiV challenge in the 2.5×10^3^ to 6.5×10^4^ pfu i.t. group (Subjects 4–8). Here, only Subject 7 had detectible viral RNA at day 7 similar to the low and variable detection of RNA observed in Subjects 1 and 3 (**Figure 6**), all other samples from Subjects 4, 5, 6, and 8 were negative (data not shown). Together, these data suggest the virus can spread in the host via a hematagenous route in the cell-free blood compartment. In almost all cases where NiV N RNA was detected, the highest RNA levels corresponded with the day of euthanasia or death of the animal. Subject 2 had an initial peak of viremia on day 10 during a period of severe clinical illness (**Table 1**), but by day 17 started to recover from disease. Interestingly, although viremia was detected again on day 21, this animal made a full recovery and was completely protected from a second NiV challenge 38 days later (data not shown). For the remaining animals, the correlation of peak viral RNA in the blood and severe illness suggests a progressive infection that seeded multiple organs simultaneously from the initial site of virus replication.

**Table 2:** Viral load in African green monkeys after Nipah virus challenge.

Subject No.	Day of death	Sample	Days after challenge							
			3	4	7	8	9	10	11	14
1	9	Plasma	0	0	2.65	NT	4.16			
2	Survived	Plasma	0	0	0	NT	NT	0	NT	0
		Whole blood	0	0	1.40	NT	NT	0	NT	0
3	11	Plasma	0	0	0	NT	0	1.40	0	
		Whole blood	0	0	0	NT	NT			
4	10	Plasma	0	0	0	NT	NT	0		
		Whole blood	0	0	0	NT	NT	0		
5	11	Plasma	0	0	0	NT	NT	0	0	
		Whole blood	0	0	0	NT	NT	0	0	
6	9	Plasma	0	0	0	NT				
		Whole blood	0	0	0	NT				
7	9	Plasma	0	0	0	NT	0			
		Whole blood	0	0	0	NT	0			
8	12	Plasma	0	0	0	NT	NT	0	NT	
		Whole blood	0	1.40	0	NT	NT	0	NT	

*Log 10 pfu of Nipah virus per ml of plasma; NT: not tested.

**Figure 7:**
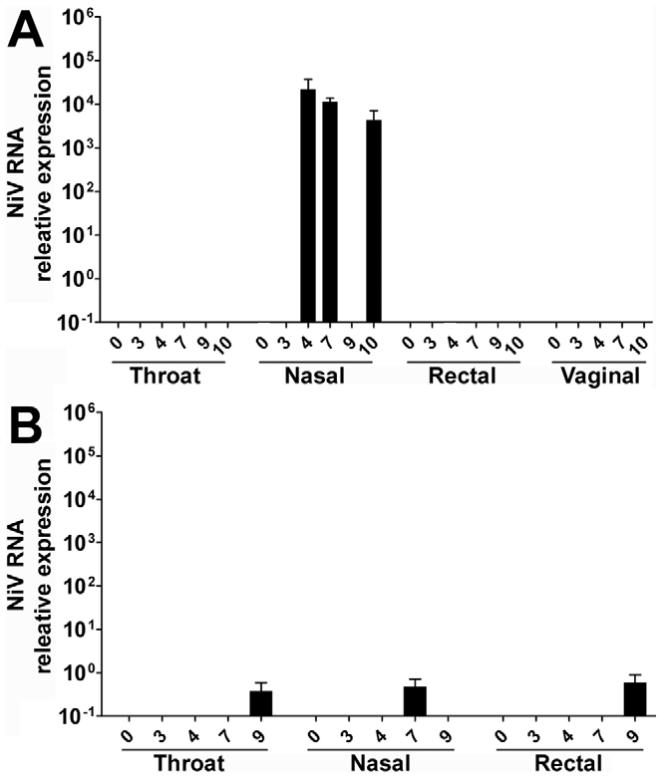
The relative quantity of NiV RNA detected by Taqman RT-PCR. Shown are the results from nasal, pharyngeal, rectal, and vaginal swabs from challenged AGM specimens at a series of time points during infection. (A) Medium challenge dose, Subject 3; (B) High challenge dose, Subject 1.

Over the course of the initial study, swab samples from the nasal passages, pharyngeal surface, rectum, and vaginal mucosa (where appropriate) were also collected and assayed. No viral RNA was detected in vaginal samples or from swab samples obtained from Subject 2 (low challenge dose). **Figure 7** shows a comparison of the relative quantity of NiV RNA detected among the remaining swab samples. Viral RNA was most often detected in nasal and throat swabs. Longitudinal periodic detection (Subject 3: **Figure 7A**) was similar to observations made in the cat model. The detection of viral RNA in Subject 1 swab samples (**Figure 7B**) was co-incident with an increased burden of virus in blood and tissue samples and severe acute disease. Together, these results indicate virus can be detected at multiple mucosal surfaces of the respiratory system.

**Figure 8:**
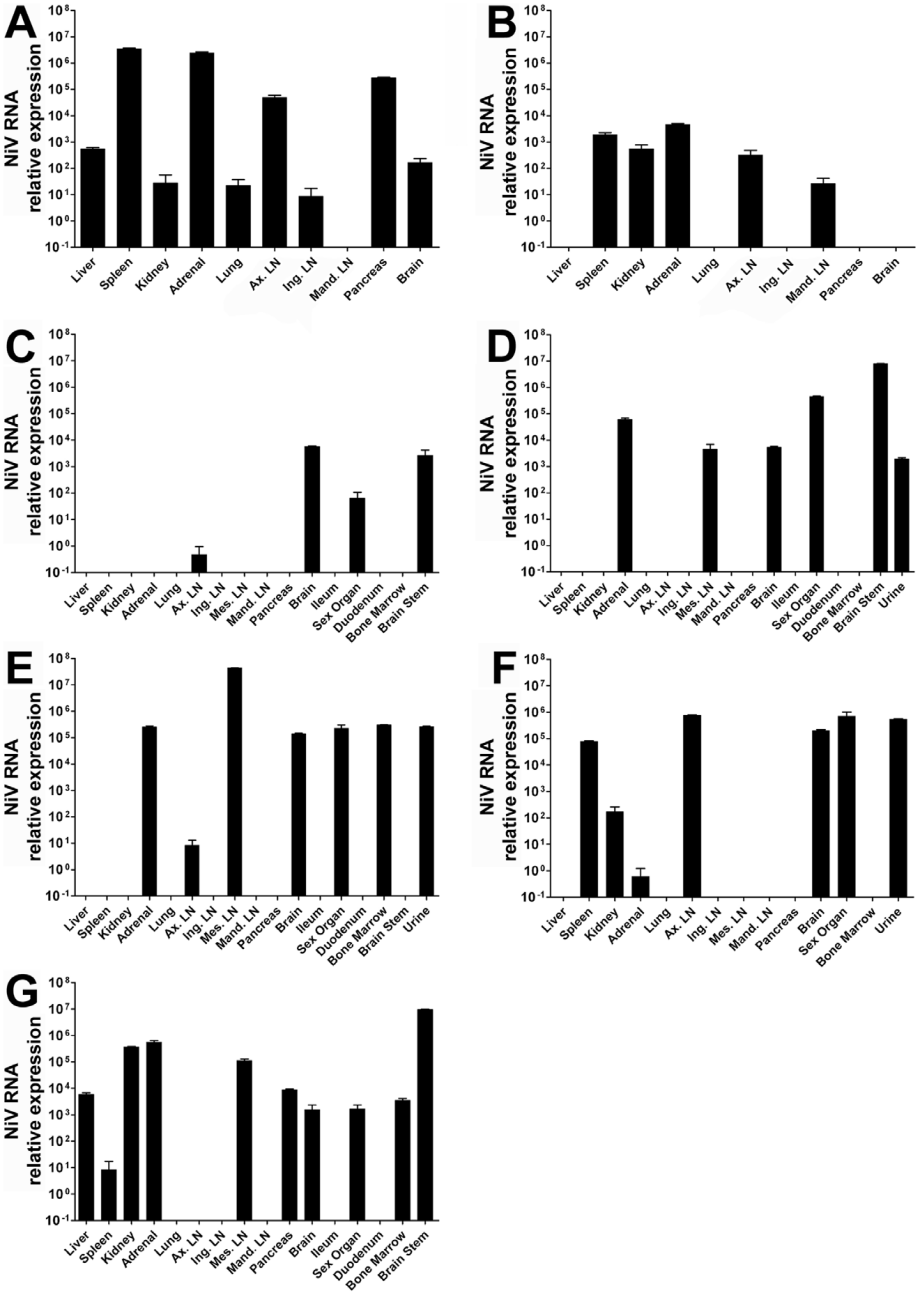
The relative quantity of NiV RNA detected by Taqman RT-PCR among AGM tissue samples obtained at necropsy from all Subjects except Subject 2 (survived). Tissue samples were stored in TriPure reagent and total RNA from each sample was isolated according to the manufacturer's directions following homogenization. (A) Subject 1; (B) Subject 3; (C) Subject 4; (D) Subject 5; (E) Subject 6; (F) Subject 7; (G) Subject 8. Abbreviations: axillary lymph node (Ax. LN), inguinal lymph node (ing. LN), mesenteric lymph node (Mes. LN), and mandibular lymph node (mand. LN).

The gross pathology and immunohistochemistry staining of tissues from the infected AGM subjects suggest spread of the virus occured rapidly (3–4 days post-infection) to numerous organ systems. Detection of viral RNA among a variety of tissue samples following necropsy (Subjects 1, 3–8) demonstrated the virus can be found in virtually every organ system sampled at the time of death depending on the animal examined (**Figure 8**). Viral RNA was highest among the spleen, adrenal gland, axillary lymph node, and pancreas. Other tissues with high levels of viral RNA included the brain, spleen and kidney. Further study is required to establish the time course of infection of these organ systems. Viral RNA was detected in only a single lung sample even though viral antigen can clearly be demonstrated in all of the lungs from challenged animals. The extensive lung pathology, severe necrosis and fibrin deposition, observed at the time of necropsy, likely contributed to the inability to detect viral RNA or isolate 18S rRNA from these sites.

## Discussion

In the present study, we investigated a new nonhuman primate model of acute NiV infection and pathogenesis using AGMs. These are the first experiments conducted in any large nonhuman primate species using a henipavirus (NiV). Unlike the previously reported NiV cat, hamster and squirrel monkey models, severe respiratory pathology, neurological disease and generalized vasculitis all manifested in NiV-infected monkeys, providing an accurate reflection of what is observed in NiV-infected humans. Here, we have observed a uniformly lethal disease with doses as low as ∼2×10^4^ pfu. A wider experiment now needs to be conducted to accurately define the minimal lethal infectious dose of NiV in this new model. Gross pathological findings revealed widespread tissue distribution of NiV with findings quite similar to those reported in NiV-infected humans, characterized as a systemic vascular disease with extensive inflammation in the respiratory epithelium associated with viral antigen and endothelial syncytia, and the brain also affected with vascular lesions.

A number of NiV RNA positive swabs from rectal, throat and nasal-oral samples were identified suggesting the possibility of virus-shedding. In addition, heavy involvement of virus infection and replication was seen in the bladder, and henipaviruses have been shown to be shed in urine. Viral shedding at the mucosal surfaces may facilitate the transmission of the virus among mammalian species in the absence of the reservoir host, including among humans. Person-to-person transmission documented during repeated epidemics in Bangladesh can potentially be explained as resulting from the aerosolization of viral particles shed at the surface of the pharynx. Future experimentation focusing on the amounts and location of infectious virus shedding will be important.

We isolated virus from subjects 1 through 5 from plasma or whole blood samples. These data indicate a low-level viremia can be detected prior to the advancement of the disease in the animal. Detection of the virus principally among plasma samples of the animals supports the PCR data in showing the virus likely disseminates to multiple organ systems from the initial site of infection by hematogenous spread in the cell-free blood component. Virus isolation from blood and plasma samples was consistently difficult when compared to the robust results detected by PCR. This phenomenon is not, however, unexpected as RT-PCR is far more sensitive than virus plaque assay. Further, detection of virus in RT-PCR is not limited to infectious particles, but also measures non-infectious particles and free viral RNA among samples. As anticipated from those observations, we also measured a wide presence of NiV RNA across a variety of tissue samples. Indeed, one of the most encouraging signs from the studies reported here was the presence of NiV RNA in neuronal tissue in conjunction with immunohistochemical staining of viral antigen in endothelial cells in the brain.

This is the first report demonstrating an acute, highly pathogenic and lethal NiV infection model in a nonhuman primate, the AGM, and provides a path for the establishment of a BSL-4-restricted primate animal model for NiV. There are currently no approved antiviral drugs or vaccines available for the henipaviruses, and neither virus productively infects or causes disease in typical small animal models, including mice or rabbits [5], [29]. Recently, the susceptibility of squirrel monkeys to NiV was examined [38]. Although some squirrel monkeys demonstrated limited similarities to NiV pathogenesis in humans only 50% of challenged animals exhibited any clinical signs with most remaining well, even following intranasal or intravenous delivery of doses as high as 10^7^ pfu of NiV. Among other smaller animal models explored, the response to either NiV or HeV in guinea pigs has been reported to be highly variable. Although a generalized vascular disease was observed with HeV in guinea pigs there was little or no pulmonary edema [39], [40]. Infection of guinea pigs with high doses of NiV (10^7^ PFU) showed only transient clinical signs followed by recovery [41] and another study using a high dose of virus by intraperitoneal administration produced disease in less than half of challenged animals [42]. There have also been some potential limitations noted in two other well-characterized animal models, the cat and hamster [24], [39], [41], [43], [44], particularly no overt CNS pathogenesis or respiratory disease, respectively. Only the recently described ferret model of NiV infection exhibited both severe respiratory and neurological disease and generalized vasculitis in which the underlying pathology closely resembled NiV-mediated disease seen in humans [28].

In summary, our findings reported here provide evidence of a new, consistent and highly pathogenic, NHP model of NiV infection that will be critical for future work on this important emerging pathogen. Based on all available reports to date, our NHP model of acute NiV infection appears to provide one of the best reproductions of human pathology observed. Together, the AGM and ferret models of NiV infection appear to be the best available platforms to comply with the “two animal rule” and will be critical in the evaluation of both passive and active immunization or therapeutic strategies for human use [45], [46].

## Materials and Methods

### Ethics Statement

Animal studies were performed in BSL-4 biocontainment at USAMRIID and were approved by the USAMRIID Laboratory Animal Use Committee. Animal research was conducted in compliance with the Animal Welfare Act and other Federal statutes and regulations relating to animals and experiments involving animals and adheres to the principles stated in the *Guide for the Care and Use of Laboratory Animals*, National Research Council, 1996 [47]. The facility where this research was conducted is fully accredited by the Association for Assessment and Accreditation of Laboratory Animal Care International.

### Virus

NiV was obtained from a patient from the 1998–99 outbreak in Malaysia [48]. NiV stocks were prepared by three passages on Vero cells (stock virus titer 1×10^7^ pfu/ml).

### Animal Studies

An environmental enrichment strategy for animals was unrestricted and provided by toys, treats, fresh fruits and vegetables. Animals were monitored daily for health, humane treatment and husbandry conditions, and acclimated to a BSL-4 animal room for a minimum of 5 days before protocol commencement. All procedures were carried out with sedated animals and all NiV-infected animals were closely monitored for signs of clinical illness and a general distress and a humane endpoint scoring system was used to determine study endpoint.

Eight henipavirus-seronegative adult African green monkeys (AGM) (*Chlorocebus aethiops*) (5–7 kg) were used for these studies. Animals were exposed to NiV by intratracheal (i.t.) or i.t. and oral routes with a range of virus doses as shown in **Table 1**. All animals were closely monitored for evidence of clinical illness (temperature, respiration, anorexia, dehydration, central nervous system disturbances, reduced activity and changes in behavior, and changes in complete blood count and chemistry) following NiV challenge. Physical exams and blood and swabs (nasal, oral, rectal) were collected before NiV challenge and at 3, 4, 7, 10, and 14, days after NiV challenge.

### Hematology and Serum Biochemistry

Total white blood cell counts, white blood cell differentials, red blood cell counts, platelet counts, hematocrit values, total hemoglobin, mean cell volume, mean corpuscular volume, and mean corpuscular hemoglobin concentration were determined from blood samples collected in vacutainer tubes containing EDTA, by using a laser-based hematologic Analyzer (Coulter Electronics, Hialeah, FL, USA). Serum samples were tested for concentrations of albumin (ALB), amylase (AMY), alanine aminotransferase (ALT), aspartate aminotransferase (AST), alkaline phosphatase (ALP), gamma-glutamyltransferase (GGT), glucose (GLU), cholesterol (CHOL), total protein (TP), total bilirubin (TBIL), blood urea nitrogen (BUN), and creatinine (CRE) by using a Piccolo Point-Of-Care Blood Analyzer (Abaxis, Sunnyvale, CA, USA).

### Sample Collection and RNA Isolation

Swab (oral, nasal, rectal, and vaginal) and blood samples were collected as described above. Tissue samples (liver, spleen, kidney, adrenal, lung, lymph node, pancreas, brain, and bone marrow) were harvested from animals at the time of necropsy. Blood samples were collected in vacutainer tubes containing EDTA and a portion of each was separated into individual blood components (plasma, granulocytes, peripheral blood mononuclear cells, and lysed red blood cells) by gradient centrifugation using Ficoll 400 (Sigma-Aldrich, St. Louis, MO) at 400×g for 45 min at room temperature. Tissue samples were ground in TriPure reagent (Roche Applied Sciences, Indianapolis, IN) at a concentration of 10% w/v. Blood samples were immediately diluted in TriPure Isolation Reagent at a ratio of 1 part plasma or swab sample to 4 parts TriPure reagent, 1 part whole blood, granulocytes, lysed red blood cells, or PBMC sample to 8 parts TriPure reagent. TriPure diluted samples were stored at −80°C. All samples were thawed at room temperature prior to RNA isolation. Total RNA was isolated from each TriPure dilute sample and each sample was resuspended with 35 µl of ultrapure water treated with 0.1% diethylpyrocarbonate (DEPC; Invitrogen, Carlsbad, CA). Samples of purified RNA were stored at −20°C.

### TaqMan PCR

TaqMan PCR was performed using the primer sets and probes as previously described [23]. Samples were amplified with a GeneAmp 7500 Sequence Detection System (Applied Biosystems). Ct values representing NiV RNA in samples were analyzed and data recorded as relative quantity or relative quantity per ml of sample.

### Virus Isolation

Virus isolation was only attempted from specimens positive for NiV RNA by PCR. Virus titration was performed by plaque assay on Vero cells as previously described [49]. Briefly, increasing 10-fold dilutions of the samples were adsorbed to Vero monolayers in duplicate wells (0.2 ml per well); thus, the limit for detection was 25 pfu/ml.

### Histopathology and Immunohistochemistry

A complete necropsy was performed on all animals. Tissue samples of all major organs were collected from each monkey for histopathologic and immunohistochemical examination and were immersion-fixed in 10% neutral buffered formalin. Replicate sections of spleen, liver, lung, kidney, axillary lymph nodes, and inguinal lymph nodes were stained with phosphotungstic hematoxylin (PTAH) to demonstrate polymerized fibrin [50]. All immunohistochemical staining was done using an immunoperoxidase-based system (Envision+, DAKO Corporation, Carpenteria, CA). Tissue sections were pretreated with a TRIS/EDTA buffer at 97°C for 30 min and blocked with 5% normal goat serum. The primary antibody was either a rabbit anti-NiV polyclonal antibody [37] diluted 1∶1000, incubated for 30 min at room temperature or a monoclonal antibody to fibrin II (NYB-T2G1, Accurate Chemical Scientific Corporation, Westbury, NY) diluted 1∶100, incubated overnight. Sections were rinsed, and then exposed to a peroxidase -labeled polymer (Envision®, DAKO) for 30 min. Color development was attained by exposing tissue to 3,3′-diaminobenzidine (DAB) for 5 min and counter-stained in hematoxylin.

### Electron Microscopy

Select tissues for ultrastructural examination were immersion-fixed in 4% formaldehyde plus 1% glutaraldehyde in 0.1 mol/L Millonig's phosphate buffer for transmission electron microscopy. Tissues were postfixed in 1% osmium tetroxide in 0.1 mol/L phosphate buffer, rinsed, stained with 0.5% uranyl acetate in ethanol, dehydrated in graded ethanol and propylene oxide, and embedded in Poly/Bed 812 resin (Polysciences, Warrington, PA). Ultrathin sections were cut, placed on 300-mesh nickel TEM grids, stained with uranyl acetate and lead citrate, and examined using a JEOL 1010 transmission electron microscope (JEOL Ltd., Peabody, MA) at 80 kV.

### X-Ray images

X-ray images were obtained using the The MinXray model HF 100/30 portable x-ray unit (Northbrook, IL) operated at 50 kV 12 mAs at 0.40 seconds at a distance of 36 inches from the subject. Fuji Type C digital 14×17 inch cassettes were employed and processed using the Fuji Smart CR® processor (Fujifilm USA, Stamford, CT).
